# Supplementary figures and images for: Atrial fibrillation as a novel risk factor for retinal stroke: A protocol for a population-based retrospective cohort study
Source: PLoS One. 2023 Dec 29;18(12):e0296251. doi: 10.1371/journal.pone.0296251 (PMC10756549; doi:10.1371/journal.pone.0296251)

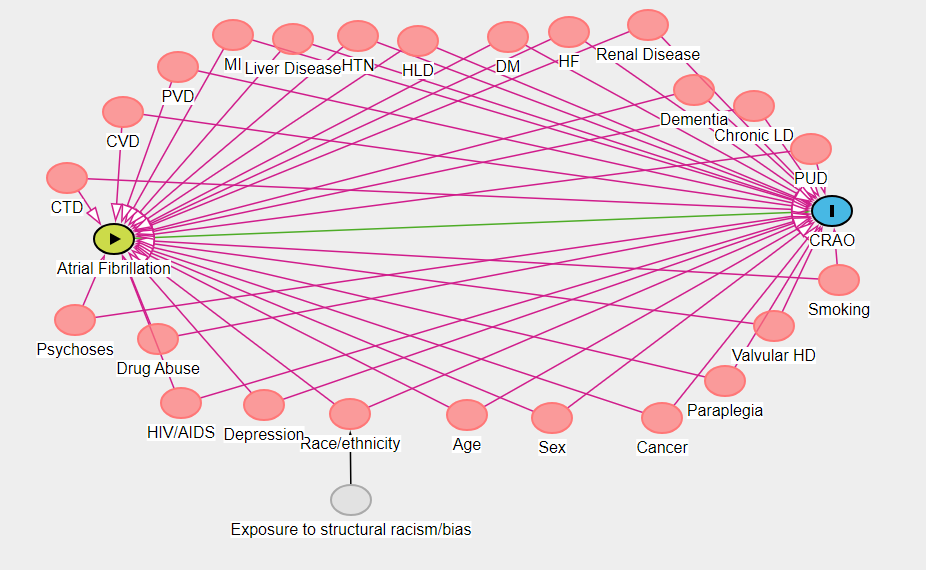

Supplement: S1 Fig — This directional acyclic graph shows our approach to addressing confounding for our first aim. (TIF) [file pone.0296251.s001.tif]

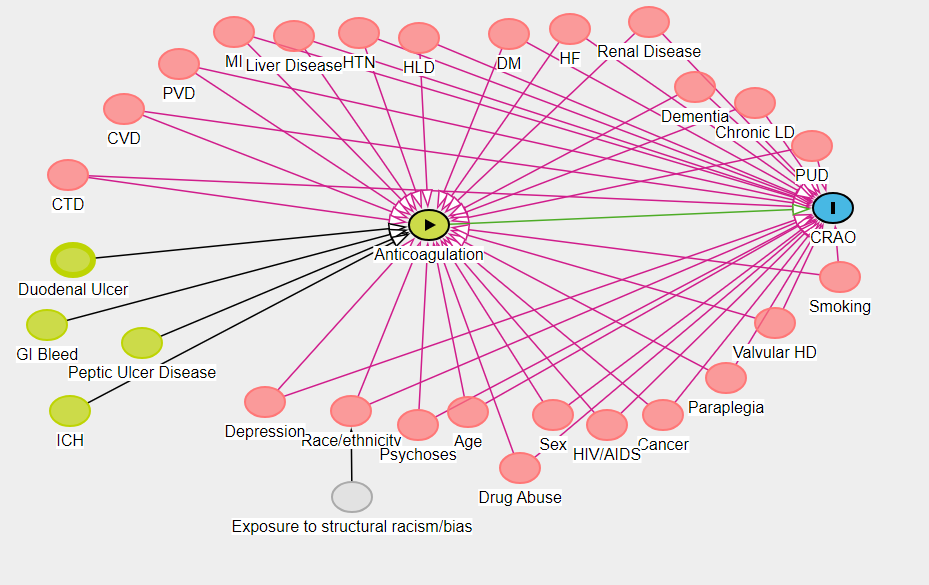

Supplement: S2 Fig — This directional acyclic graph shows our approach to addressing confounding for our second aim. (TIF) [file pone.0296251.s002.tif]
