# Supplementary material for: Atrial fibrillation as a novel risk factor for retinal stroke: A protocol for a population-based retrospective cohort study
Source: PLoS One. 2023 Dec 29;18(12):e0296251. doi: 10.1371/journal.pone.0296251 (PMC10756549; doi:10.1371/journal.pone.0296251)
Supplement: Attachment — Submitted filename: Response to Reviewers.docx [file pone.0296251.s004.docx]

**RESPONSE TO REVIEWERS**

**The authorship team would like to thank the scientific reviewer for their thoughtful and constructive comments. These have greatly enriched the manuscript. Please see below our point-by-point responses below.**

**1- Sample size calculation: Indicate the statistical testing method which attains 90% power.**
-Thank you for this comment. We apologize for this oversight. We have addressed with the following addition:

“Power/sample size calculations were made on the basis of an unadjusted Cox proportional hazards regression model.”

Please note that we felt that an unadjusted mode as the basis for our power calculations was appropriate because we intend to first perform a matching procedure and further covariate adjustment is not anticipated to impact our results.

**2- Descriptive analysis: Perhaps the statement, “We will summarize count data using . . .” can be clarified by stating, “We will summarize categorical data using . . .”**
-We appreciate this comment. I have re-written this section accordingly.

**3- Tables 7 and 8: Clarify that the “Unadjusted Model” reports univariate results whereas the “Adjusted Model” reports multivariate results.**
-Thank you very much for this comment. We have updated these tables to ensure this point is conveyed:

“†This column reflects the results of a model that incorporates overlap weighting using propensity scores re-estimated after our first stage matching procedure”

**4- Below Table 7: Indicate the statistical method that will be used to graphically displaying the adjusted cumulative incidence of CRAO.**
-Thank you very much. We have added the statistical method:
“We will report a figure showing the adjusted cumulative incidence of CRAO according to AF vs. No AF groups using an overlap-weighted cumulative incidence function.”

**5- Identify the software that will be used for the statistical analysis.**
-Thank you for this comment. We have updated this as follows:
“**Analytic Software**

All analyses will be performed using SAS Version 9.4 (Cary, NC).”

**6- Provide details about the characteristics on which the matched controls will be selected.**
-Thank you very much for this comment. We have provided the following details about the characteristics on which the matched controls will be selected:

“All remaining beneficiaries will be considered potential controls; inclusion and exclusion criteria will be applied based on an index date of July 1 for each year in the study period; all beneficiaries meeting inclusion/exclusion criteria for each year will be included in the potential control pool. For all potential controls with multiple index dates/years of eligibility, the year they contribute will be selected randomly. A multivariable logistic regression analysis will be conducted using cases and potential controls to create propensity scores for AF status; the final control group will be selected using propensity score matching to the AF cases.”

**7- To assist in the review process, add line numbers to the document.**

Thank you, we have made this change.
